# Molecular heterogeneity assessment by next-generation sequencing and response to gefitinib of *EGFR* mutant advanced lung adenocarcinoma

**DOI:** 10.18632/oncotarget.3727

**Published:** 2015-03-30

**Authors:** Emilio Bria, Sara Pilotto, Eliana Amato, Matteo Fassan, Silvia Novello, Umberto Peretti, Tiziana Vavalà, Stefania Kinspergher, Luisella Righi, Antonio Santo, Matteo Brunelli, Vincenzo Corbo, Eliana Giglioli, Isabella Sperduti, Michele Milella, Marco Chilosi, Aldo Scarpa, Giampaolo Tortora

**Affiliations:** ^1^ Department of Medicine, Medical Oncology, University of Verona, Azienda Ospedaliera Universitaria Integrata, Verona, Italy; ^2^ ARC-NET Center for Applied Research on Cancer, University and Azienda Ospedaliera Universitaria Integrata, Verona, Italy; ^3^ Department of Oncology, University of Torino, A.O.U. San Luigi, Orbassano, Torino, Italy; ^4^ Department of Pathology and Diagnostics, University and Azienda Ospedaliera Universitaria Integrata, Verona, Italy; ^5^ Biostatistics, Regina Elena National Cancer Institute, Rome, Italy; ^6^ Medical Oncology, Regina Elena National Cancer Institute, Rome, Italy

**Keywords:** lung cancer, EGFR, next-generation sequencing, gefitinib

## Abstract

Cancer molecular heterogeneity might explain the variable response of *EGFR* mutant lung adenocarcinomas to tyrosine kinase inhibitors (TKIs). We assessed the mutational status of 22 cancer genes by next-generation sequencing (NGS) in poor, intermediate or good responders to first-line gefitinib. Clinical outcome was correlated with Additional Coexisting Mutations (ACMs) and the *EGFR* Proportion of Mutated Alleles (PMA). Thirteen ACMs were found in 10/17 patients: *TP53* (n=6), *KRAS* (n=2), *CTNNB1* (n=2), *PIK3CA*, *SMAD4* and *MET* (n=1 each). *TP53* mutations were exclusive of poor/intermediate responders (66.7% *versus* 0, p=0.009). Presence of ACMs significantly affected both PFS (median 3.0 *versus* 12.3 months, p=0.03) and survival (3.6 months *versus* not reached, p=0.03). *TP53* mutation was the strongest negative modifier (median PFS 4.0 *versus* 14.0 months). Higher *EGFR* PMA was present in good *versus* poor/intermediate responders. Median PFS and survival were longer in patients with *EGFR* PMA ≥0.36 (12.0 *versus* 4.0 months, p=0.31; not reached *versus* 18.0 months, p=0.59). Patients with an *EGFR* PMA ≥0.36 and no ACMs fared significantly better (p=0.03), with a trend towards increased survival (p=0.06). Our exploratory data suggest that a quantitative (PMA) and qualitative (ACMs) molecular heterogeneity assessment using NGS might be useful for a better selection of patients.

## INTRODUCTION

Randomized clinical trials conducted in non-small-cell lung cancer (NSCLC) carrying activating mutations of the epidermal growth factor receptor (*EGFR*) have clearly shown that tyrosine kinase inhibitors (TKIs) dramatically contribute to improve prognosis, disease control, symptoms and quality of life when compared to traditional platinum-based chemotherapy [1-5]. A recent meta-analysis confirmed that *EGFR* mutant NSCLC patients derived a significant progression-free-survival (PFS) advantage from TKIs over platinum-doublet chemotherapy as first-line treatment, although a significant differential benefit may be observed according to smoking status (HR for never-smokers 0.29 *versus* 0.54 for ever-smokers; *p* < 0.007) and to the type of *EGFR* mutation (HR for exon 19 deletion 0.25 *versus* 0.44 for exon 21 substitution; *p* < 0.001) [6].

Therefore, the anti-*EGFR* TKIs gefitinib, erlotinib and afatinib are currently employed for the treatment of patients with advanced lung adenocarcinoma harboring *EGFR* activating mutations. However, the duration of response is variable and almost 25% of patients rapidly progress during treatment, many at the first disease assessment time-point. The question arises whether there are additional candidate clinical and/or molecular predictive factors permitting a further selection (*‘super-selection’*) of patients with *EGFR* mutated cancers to implement prediction of the awaited TKIs efficacy and identify those patients not beneficiating from TKIs despite the presence of the *EGFR* alteration.

The onset of resistance seems to represent an inevitable consequence of targeted therapies in solid tumors. Classically, the resistance develops after an initial response to therapy (acquired resistance) and may be pharmacological (failure of delivery of the drug to its target) or biological, primarily deriving from the activation of coexisting pathways, bypassing the oncogenic dependency of a given driven alteration [7].

Although the widely validated role of the *EGFR T790M* mutation as the main mechanism of acquired resistance to erlotinib and gefitinib [8, 9], the reliable impact of this genetic alteration at diagnosis is still debatable, relying on contradictory data regarding its true incidence and clinico-biological role. The rate of pretreatment *T790M* mutation is strongly dependent on the sensitivity of the detection method (ranging from 2% to 35%) [10, 11]. Recently, *Costa et al.* detected a high frequency (65%) of coexisting *EGFR T790M* before treatment, using a highly sensitive method based on laser microdissection and peptide-nucleic acid-clamping PCR [12]. The presence of a pretreatment *T790M EGFR* mutation seems to be associated with worse clinical outcomes (in term of objective response and PFS) to *EGFR* TKIs compared with patients with classic *EGFR* activating alterations without any detected *T790M* mutation [13, 14].

Other genetic abnormalities and signaling pathways are currently under investigation because of their potential implication in the development of TKIs resistance. *TP53* represents the most frequently mutated gene in lung cancer, occurring in over half of adenocarcinoma, 80% of squamous cell carcinoma and 70% of small-cell-lung cancer [15]. Although the prognostic role of *TP53* is still debatable, some preclinical data seem to suggest an intriguing predictive influence of *TP53* mutation. In this regard, the persistent *STAT3* activation has been observed in the residual survivor lung cancer cells both *in vitro* and *in vivo* under targeted TKIs, suggesting that early *STAT3* phosphorylation may represent an important transcriptional programming event prior to the resurgence of resistant tumor survivors [16]. Moreover, *TP53* and *PTEN* knowkdown synergize to activate pro-inflammatory interleukin-6/*STAT3*/nuclear factor kB signaling generating highly metastatic epithelial-to-mesenchymal transition-like cancer stem cells. The constitutive activation of this loop leads to the suppression of *SOCS3* (suppressor of cytokine signaling 3), a critical negative regulator of pro-inflammatory pathways, suggesting interesting connection between inflammation and carcinogenesis [17].

Several clinical approaches can help to maintain the disease control in the resistance setting, including the use of radiation to treat isolated areas of progression (classically the central nervous system) and switching to cytotoxic chemotherapy. Moreover, novel approaches have already demonstrated a strong signal of activity, such as the development of second-generation and third-generation inhibitors and the combination of some of these inhibitors with antibodies directed against the same target. In this continuously evolving setting, the increased understanding of the spectrum of resistance is mandatory to make progress in clinical research [18].

Recently, many new facets emerged, highlighting the fact that *EGFR* mutations may be potentially targeted even with drugs without a peculiar *EGFR* action, such as bisphosphonates [19] and ibrutinib (a Bruton tyrosine kinase and *BMX* inhibitor) [20], that have demonstrated activity in *EGFR*-mutant NSCLC cell lines, including erlotinib-resistant tumors. Other promising data suggest the fact that the *EGFR* mutation may represent a genetic biomarker predicting enhanced sensitivity to topoisomerase II inhibitor (such as etoposide), in response to a methyltransferase *EZH2* inhibitor, supporting the biological rationale underlying the possibility of a combined approach with these molecules [21]. With regard to the hypothesis of a multi-targeted approach, another recent preclinical analysis demonstrated that adding chloroquine (acting as an autophagy inhibitor) to *EGFR* and *AKT* inhibition might potentially improve tumor responses in *EGFR* mutant NSCLC cells [22]. Several experimental approaches, such as the establishment of pooled short-hairpin RNAs library screen, are currently under investigation to identify promising drugs and pathways for further study in *EGFR* mutant NSCLC, including TKIs-resistant NSCLC [23].

Tumor heterogeneity strongly contribute to primary resistance. The demonstration that human cancers are frequently represented by a molecular mosaic of cells, spatially and temporally different, derived from several genomic studies (reviewed in [24]). Next-generation sequencing approaches in renal and pancreatic cancers have demonstrated the existence of a strong heterogeneity among different regions within the same tumor and between the primary tumor and metastasis [25, 26].

The codification of tumor heterogeneity, improving the understanding of the oncogenic mechanism inclusive of the totality of the genomic and epigenomic processes, may help to identify clinically relevant subgroups of patients, leading to a better management of the therapeutic resistance to targeted agents.

In this context, the assessment of molecular heterogeneity with the innovative multigene next generation sequencing (NGS) technology may help to concurrent screen for additional genetic abnormalities potentially deputed to drive cancer predictive testing for therapeutic decisions [27]. The implications for future research are challenging: the *EGFR* mutated dosage as well as coexisting mutations could become a new predictive tool for lung cancer patients and new technologies, such as NGS, may potentially be introduced in routine practice.

## RESULTS

Eighteen patients from two institutions, whose characteristics are reported in Table 1, were studied. Median age was 71 years (range 37-83). Smokers were significantly more represented in the poor group than in the others (66.7% *versus* 10.0%, p[Fisher]=0.04). Seventeen patients were evaluable for PFS at least at first evaluation. At a median follow-up of 8 months (range: 1-33), 14 events of progression and 7 deaths were recorded. None of the patients had rearrangements of the *ALK* gene. Six, 3 and 8 patients were grouped as poor, intermediate and good responders, respectively, according to the treatment resistance to gefitinib and PFS (Table 2). The median PFS was 1.7, 6.1 and 17.3 months for poor, intermediate and good responders, respectively (*p* < 0.0001, Table 2).

**Table 1:** Patients' characteristics (overall patients' sample)

Patients' characteristic	[n] (%)
Sex	
Male	6 (33.3)
Female	12 (66.7)
ECOG Performance Status	
0	14 (77.8)
1	4 (22.2)
Smoking status	
Current	6 (33.3)
Never smokers	12 (66.7)
Stage	
Recurrent	8 (44.4)
IIIB	2 (11.2)
IV	8 (44.4)
Number of metastatic sites	
0	6 (33.4)
1	8 (44.4)
2	2 (11.1)
≥3	2 (11.1)
Brain metastases	
Yes	4 (22.2)
No	14 (77.8)
Previous surgery	
Yes	8 (44.4)
No	10 (55.6)
Diagnostic procedure	
FNAB	1 (5.6)
Thoracic biopsy	8 (44.4)
Surgical specimen	8 (44.4)
Other extra-thoracic biopsy	1 (5.6)
EGFR mutation	
Exon 19 Deletion	13 (72.2)
L858R	5 (27.8)
Response to treatment	
Objective Response	12 (66.7)
No response	6 (33.3)

n, number of patients; ECOG, Eastern Cooperative Oncology Group; FNAB: fine needle aspiration biopsy; EGFR: epidermal growth factor receptor.

**Table 2:** Patients' groups according to resistance to Gefitinib and Progression-Free-Survival; 17 evaluable patients (Log-Rank p < 0.0001)

Group	Definition	Pts (%)	Median PFS (months, 95%CI)
Poor	Progression at 1^st^ assessment	6 (35.2)	1.7 (0.1-3.2)
Intermediate	Progression within 12 months	3 (17.7)	6.1 (3.0-9.2)
Good	Progression ≥ 12 months or treatment ongoing	8 (47.1)	17.3 (9.0-25.5)

Pts: patients; PFS: progression-free-survival; CI: confidence intervals.

The proportion of cancer cells carrying mutated EGFR was calculated on the basis of the output of NGS analysis that furnishes the proportion of the mutated alleles (PMA), i.e., the number of mutated alleles over the total alleles analyzed. Thus, as the alleles in a cell are two, the proportion of cancer cells carrying a mutated allele is generally the double of the PMA indicated by NGS analysis, and referred to a cancer cellularity of at least 60% obtained through microdissection. As for *EGFR* mutations, median PMA was 0.2 (range 0.12-0.36), 0.16 (range 0.15-0.65) and 0.35 (range 0.17-0.61) for poor, intermediate and good responders, respectively. A non-significant trend between the good responder group and the 2 others was found in median PMA (0.35 *versus* 0.21, p[t-student]=0.08; p-value[Mann-Whitney]=0.16). A rate of 0.36 for PMA was identified at the ROC analysis as the best cut-off to split patients according to PFS.

**Figure 1:**
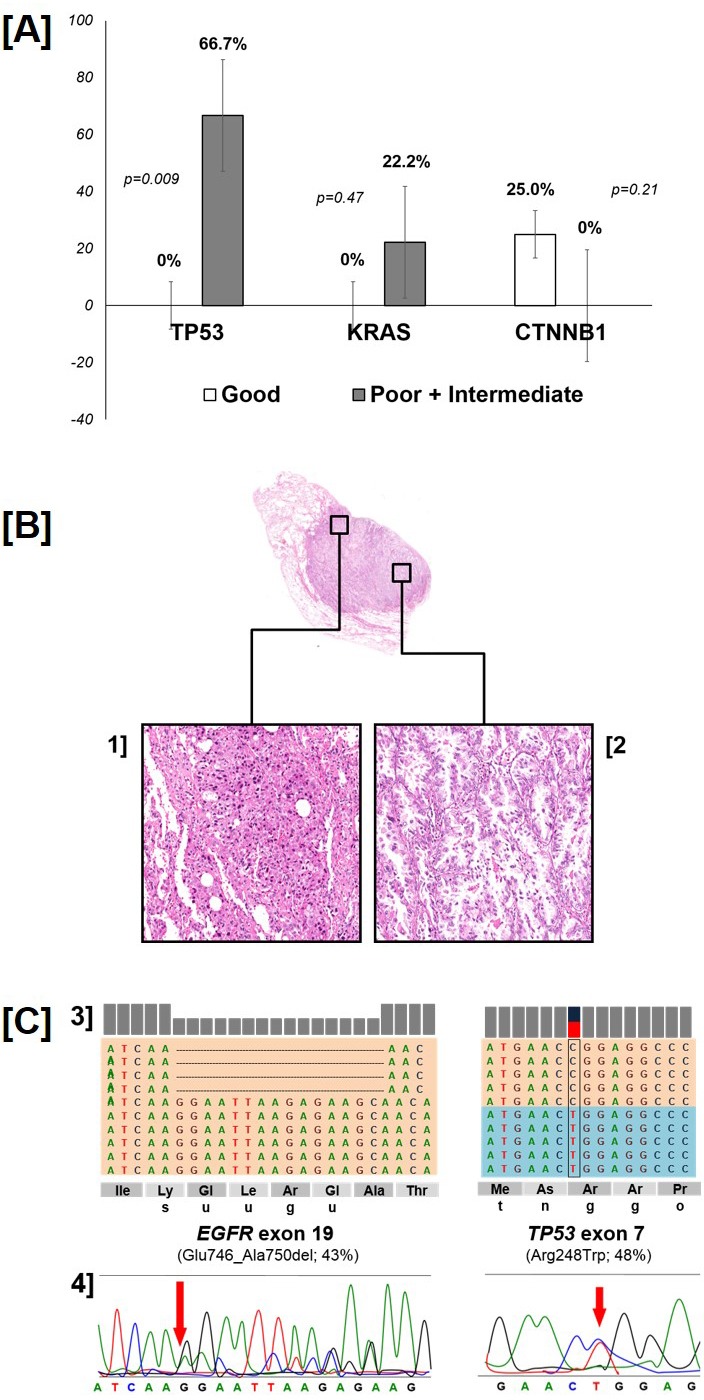
Distribution of *TP53*, *KRAS* and *CTNNB1* gene mutation according to group (p-value: Fisher's exact test - panel A) A representative case of intratumor histologic and molecular heterogeneity (panel **B-C**). The poor responder case presented well-differentiated coexisting with de-differentiated areas within the same specimen (panel **B**; original magnifications 4x and 20x). Of interest, an *EGFR* deletion in exon 19 was observed in the well-differentiated adenocarcinoma, that was associated with a concomitant *TP53* mutation (*R248W*) in the more de-differentiated area. The representation of the reads obtained by Ion Torrent sequencing, aligned to the reference genome as provided by the Integrative Genomics Viewer (IGV v.2.1, Broad Institute) software for the mutations in *EGFR* and *TP53* genes, and the corresponding Sanger sequencing are reported. (panel **C**).

Thirteen mutations in addition to the one in *EGFR* were found in the *TP53* (n=6), *KRAS* (n=2), *CTNNB1* (n=2), *PIK3CA*, *SMAD4* and *MET* (n=1 each) gene. All mutations were confirmed by Sanger sequencing. None of the patients had more than 2 concomitant mutations. The association of additional mutations and prognostic groups are reported in Table 3 and Supplementary Table 1. *TP53* mutations (median PMA 0.45) were exclusively found among poor and intermediate patients and lacked in good responders (66.7% versus 0%, p[Fisher]=0.009) (Figure 1, Panel A).

**Table 3:** Relative frequency of gene mutations according to patients' groups (17 evaluable patients)

Gene Analysed	Good [n] (%)	Intermediate [n] (%)	Poor [n] (%)	p-value
**TP53**				
Mutant	0 (0)	3 (100)	3 (50)	***0.005***
Wild-Type	8 (100)	0 (0)	3 (50)	
**KRAS**				
Mutant	0 (0)	0 (0)	2 (33.3)	*0.12*
Wild-Type	8 (100)	3 (100)	4 (66.7)	
**CTNNB1**				
Mutant	2 (25)	0 (0)	0 (0)	*0.28*
Wild-Type	6 (75)	3 (100)	6 (100)	
**PIK3CA**				
Mutant	0 (0)	0 (0)	1 (16.7)	*0.38*
Wild-Type	8 (100)	3 (100)	5 (83.3)	
**MET**				
Mutant	1 (12.5)	0 (0)	0 (0)	*0.55*
Wild-Type	7 (87.5)	3 (100)	6 (100)	
**SMAD4**				
Mutant	1 (12.5)	0 (0)	0 (0)	*0.55*
Wild-Type	7 (87.5)	3 (100)	6 (100)	

n, number of patients; p-value: chi-square test.

A paradigmatic case of a poor responder patient with a clear molecular heterogeneity is shown in Figure 1 (panels B-C) where an *EGFR* deletion in exon 19 in an area of the lesion presenting a well-differentiated adenocarcinoma (panels B-2 and C-3) is associated with a concomitant *TP53* mutation (*R248W*) in a more dedifferentiated area (panels B-1 and C-3); both mutations were confirmed by Sanger Sequencing (Figure 1, Panel C-4).

**Figure 2:**
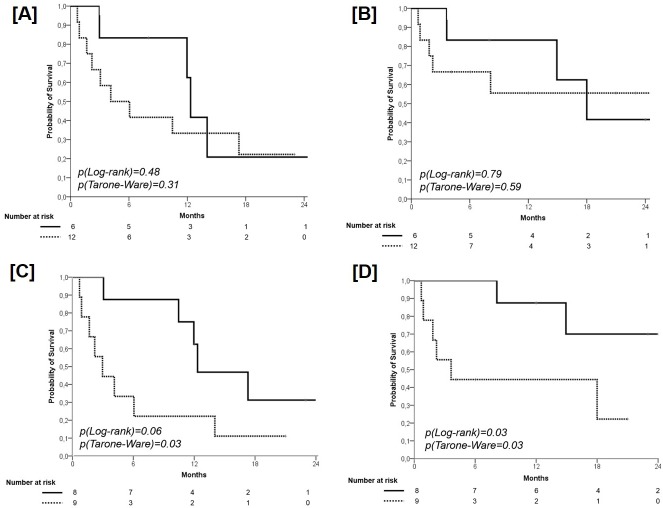
Progression-Free Survival according to PMA (cut-off 0.36) Solid line: patients with PMA ≥ 0.36; dashed line: patients with PMA < 0.36 (panel **A**). Overall Survival according to PMA (cut-off 0.36). Solid line: patients with PMA ≥ 0.36; dashed line: patients with PMA < 0.36 (panel **B**). Progression-Free Survival according to the presence of additional coexisting mutations (ACM). Solid line: patients with ACM = 0; dashed line: patients with ACM ≥ 1 (panel **C**). Overall Survival according to the presence of additional coexisting mutations (ACM). Solid line: patients with ACM = 0; dashed line: patients with ACM ≥ 1 (panel **D**).

No significant difference according to PMA in term of PFS was found (PMA ≥ 0.36, median PFS 12.0 months, 95% CI 11-13; 1-year PFS 62.0%, 2-year PFS 22.2% *versus* PMA < 0.36, median PFS 4.0 months, 95% CI 1-9; 1-year PFS 33.0%, 2-year PFS 20.8%; p=0.31), with a HR of 1.53 (95% CI 0.47-5.01, p=0.48) (Figure 2, Panel A). With regard to OS, no significant difference according to PMA was found (PMA ≥ 0.36, median OS 18 months, 95% CI 11-24; 1-year OS 83.5%, 2-year OS 41.7% *versus* PMA < 0.36, median OS not reached; 1-year OS 56.0%, 2-year OS 55.6%; p=0.59), with a HR of 1.21 (95% CI 0.28-5.16, p=0.79) (Figure 2, Panel B).

A borderline significant difference in favor of the 8 patients with ACM=0 (median PFS 12.3 months, 95% CI 6.1-18.6; 1-year PFS 61.5%, 2-year PFS 31.3%) in comparison with those 9 with a ACM≥1 (median PFS 3.0 months, 95% CI 0.7-5.3; 1-year PFS 22.0%, 2-year PFS 11.1%) was found (p=0.03), with a HR of 2.88 (95% CI 0.92-9.02; p=0.068) (Figure 2, Panel C). With regard to OS, a significant difference in favor of the 8 patients with ACM=0 (median OS not reached, 1-year OS 88.0%, 2-year OS 70.0%) in comparison with those 9 with a ACM ≥ 1 (median OS 3.6 months, 95% CI 0.0-7.8; 1-year OS 43.5%, 2-year OS 22.2%) was found (p=0.03), with a HR of 5.07 (95% CI 0.99-26.03; p=0.052) (Figure 2, Panel D).

**Figure 3:**
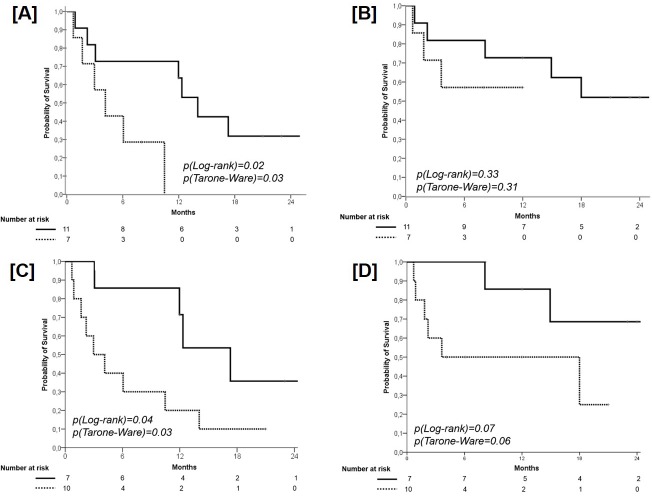
Progression-Free Survival according to the *TP53* Mutation Solid line: patients with wild type *TP53*; dashed line: patients with mutant *TP53* (panel **A**). Overall Survival according to the *TP53* Mutation. Solid line: patients with wild type *TP53*; dashed line: patients with mutant *TP53* (panel **B**). Progression-Free Survival according to the combination of PMA (cut-off 0.36) and the presence of additional coexisting mutations (ACM). Solid line: patients with PMA ≥ 0.36 and ACM = 0; dashed line: patients with PMA < 0.36 and ACM ≥ 1 (panel **C**). Overall Survival according to the combination of PMA (cut-off 0.36) and the presence of additional coexisting mutations (ACM). Solid line: patients with PMA ≥ 0.36 and ACM = 0; dashed line: patients with PMA < 0.36 and ACM ≥ 1 (panel **D**).

A significant difference in favor of the 11 patients with wild type *TP53* (median PFS 14 months, 95% CI 11.0-17.0; 1-year PFS 62.5%, 2-year PFS 31.8%) in comparison with those 7 with a mutant *TP53* (median PFS 4.0 months, 95% CI 1.0-7.0; 1-year PFS 0%) was found (p=0.02), with a HR of 4.66 (95% CI 1.12-19.37; p=0.03) (Figure 3, Panel A). With regard to OS, no significant difference according the presence of p53 status was found (wild type *TP53*, median OS not reached; 1-year OS 71.7%, 2-year OS 52.0% *versus* mutant *TP53*, median OS not reached; 1-year OS 58.0%, 2-year OS 57.0%; p=0.31), with a HR of 2.25 (95% CI 0.43-5.16, p=0.34) (Figure 3, Panel B).

A significant difference in favor of the 7 patients with an *EGFR* PMA ≥ 0.36 and ACM=0 (median PFS 17.3 months, 95% CI 11.5-23.1; 1-year PFS 70.5%, 2-year PFS 35.7%) in comparison with those 10 with an *EGFR* PMA < 0.36 and ACM≥1 (median PFS 3.0 months, 95% CI 1.0-6.0; 1-year PFS 19.4%, 2-year PFS 10%) was found (p=0.03), with a HR of 3.26 (95% CI 0.78-10.85, p=0.054) (Figure 3, Panel C). With regard to OS, a trend towards significance in favor of patients with an PMA ≥ 0.36 and ACM=0 (median OS not reached; 1-year OS 86.0%, 2-year OS 68.6%) in comparison with patients with an *EGFR* PMA < 0.36 and ACM ≥ 1 (median OS 4.0 months, 95% CI 1-18; 1-year OS 50.5%, 2-year OS 25.0%) was found (p0.06), with a HR of 4.01 (95% CI 0.78-20.59, p=0.10) (Figure 3, Panel D).

## DISCUSSION

Despite exploratory and unpowered for conclusive interpretations, the results reported herein indicate that the application of NGS technology may furnish a baseline genetic portrait of advanced NSCLC that gives information on the presence of actionable and additional mutations that may impair the efficacy of targeted therapies. A prospective validation in a large set of patients is required to corroborate our results.

Our study on a series of *EGFR* mutant advanced lung cancers receiving 1^st^ line Gefitinib suggests that the presence of ACM significantly decreases the expected benefit of TKIs.

Among the identified ACMs, *TP53* mutations were exclusively documented among poor/intermediate responders than in good responders (66.7% *versus* 0%, p=0.009), suggesting the potential implication of this gene in influencing the chance of response to the anti-*EGFR* agents. In lung cancer, *TP53* represents the most frequently mutated gene, occurring in over half of adenocarcinoma, 80% of squamous cell carcinoma and 70% of small-cell-lung cancer [15], and, despite debated [28], it has been suggested as an independent marker for shorter survival in advanced NSCLC. A recent evidence demonstrated that not all *TP53* mutations are equal: non-disruptive *TP53* mutations (those preserving some functional properties of the protein) represents an independent prognostic factor of shorter survival in advanced NSCLC (13.3 *versus* 24.6 months; HR=2.08; *p* < 0.001) [29].

In contrast to *EGFR*-activating mutations, *KRAS* mutations are usually detected in smokers, and associated with poor prognosis and no benefit from TKIs and adjuvant chemotherapy. However, some evidences are available regarding the coexistence of *KRAS* and *EGFR* mutations, raising questions about the relative values of these genetic abnormalities as predictors of outcome in NSCLC [30]. *Marchetti et al.* reported that patients carrying both mutations were resistant to TKIs and showed a shorter survival compared with those patients with only *EGFR* mutations, suggesting that the biological power determined by the presence of *KRAS* mutation, even if only in minor cellular clones, may potentially overcome the dependence to *EGFR* [31].

*EGFR* mutations are very uncommon among smokers and former smokers, enforcing the rationale that coexisting mutations may feature the disease. From a purely speculative point-of-view, we observed that 4 out of 6 *TP53* mutations, *KRAS* mutations (n=2) and the *MET* mutation (n=1) have been detected in the smaller group of *EGFR* mutant patients that were smoker, suggesting a potential correlation between smoking status and the presence of ACMs.

Our data may contribute to generate hypotheses with regard to the biological intrinsic difference between primary and acquired resistance, being the latest event supported by a series of data indicating the *EGFR T790M* mutation as the more common mechanism of acquired resistance to erlotinib and gefitinib [8]. Indeed, approximately 50-80% of rebiopses of *EGFR*-mutant patients progressing under the selective pressure of *EGFR* TKIs display this kind of mutation [8, 9]. Globally considered, our results support the hypothesis that *TP53* (and perhaps other tumor suppressor genes) may affect the efficacy of the traditional target therapy in molecularly addicted patients, thus triggering cell proliferation stimulus, and bypassing the oncogenic power of the *EGFR* pathway.

In this population carrying ‘bad’ genetic alterations, concurrently with the classical *EGFR*-activating mutations, the employment of a combination strategy of chemotherapy and target agents may preserve a clinico-biological rationale. Although no benefit was demonstrated for adding TKIs to chemotherapy (INTACT-1 and 2 [32, 33], TRIBUTE [34] and TALENT trials [35]), intercalated erlotinib to cisplatin or carboplatin demonstrated a significant benefit in the context of an *EGFR* mutation-positive subgroup (FASTACT-1 and 2) [36, 37], although these evidences warrant to be replicate in the context of non-asian patients' populations.

In addition to this ‘*qualitative’* analysis exploring the number of ACM, we tried to determine if the ‘*quantitative’* analysis of the *EGFR* mutation (PMA) might have a role in influencing the efficacy of TKIs. These data allow speculating upon the exploration of those key processes critical to the tumor development and progression, as the tumor heterogeneity.

With regard to the ‘*quantitative’* analysis of the *EGFR* mutation, *Zhou et al.* firstly hypothesized that the quantification of *EGFR* mutations might predict the extent of *EGFR* TKIs benefit, demonstrating an advantage in term of PFS for those patients with high abundance of *EGFR* mutations (detected concurrently with two methods with a different sensitivity) [38]. Our results support this hypothesis, although, given the limited number of patients, only a weak trend in favor of those patients with a high PMA (≥ 0.36) was found.

Nevertheless, given the intrinsic differences in the adopted *EGFR* detection methods, the reliability of the observed evidences and their overall conclusions must be considered purely speculative for further research in this regard. Indeed, as recently demonstrated by *Tseng et al.*, a significant portion of responses to erlotinib in *EGFR wild-type* patients was related to the limitations of detection methods (both direct sequencing and mutant type-specific sensitive methods) [39].

Although no detailed study on tumor heterogeneity have been published in lung cancer, several data regarding the complexity of the molecular background of lung cancer are available. From the morphological perspective, the most recent clinico-pathological classification of lung adenocarcinoma identifies multiple histologic subtypes, frequently co-existing in the same tumor [40]. From the genomic standpoint, recent data demonstrate the high mutational rate of lung cancer, supporting the hypothesis of a strong heterogeneity [41, 42]. Nevertheless, a series of data suggest that the mutational load of lung adenocarcinoma in non-smoker patients (where the chance of detecting the *EGFR* mutation is the highest) is significantly lower [43], and that few (but critical) mutations may be enough to switch cells from normal to malignant [44].

While conflicting evidences exist regarding the discordance rate of *EGFR* mutation status between primary tumors and metastases, a substantial consistency supports the lack of a reliable heterogeneity of *EGFR* mutations at the intratumor level [24]. Recently, *Wright et al.* analyzed different areas in the context of a series of lung adenocarcinoma specimens and found that *KRAS* and *BRAF* mutations were confined to high-grade morphological domains, while the *EGFR* mutations were identifiable through all histological subtypes in the tumor according to the driver status of the mutations [45].

Globally considered, these results suggest that the heterogeneity of a driver-gene mutation, such as that of *EGFR* in lung cancer is a rare molecular event. However, the alterations in the target gene itself (affecting the delivery of the drug to its target) and the concurrent activation or suppression of other signaling pathways (relevant for tumor progression and survival), may justify the heterogeneity in term of the clinical benefit deriving from the employment of target agents.

The data emerging from our analysis support the potential role of the NGS technology in the deeper analysis of the molecular background, also in the context of a recognized oncogene-addicted disease, with the ideal aim to further improve the *EGFR* mutant patients' selection. Our group already highlighted the basic contribution of NGS technique in the development of the translational research in the diagnostic field, able to provide, with limited amounts of the biological material (DNA), reliable quantitative and qualitative data, critical for the application of the tailored medicine [46].

Together with other emerging scientific evidences, our analysis may represent a proof-of-principle study supporting the existence of a tumor heterogeneity which, from now on, should be considered clinically relevant and deserving to be deepened for clinical validation [47]. In this new era of precision medicine, our study highlights the potential strong contribution to the understanding of the molecular bases of cancer yielded by NGS technologies, which may allow to provide a ‘*qualitative’* (presence of ACM) and ‘*quantitative’* (PMA) measure of the tumor heterogeneity [48]. In this regard, the found genetic portrait of cancer cells may mirror the underlying tumor heterogeneity, thus allowing to ultra-stratifying those *EGFR* mutant NSCLC patients, and estimating a differential clinical benefit of anti-*EGFR* TKIs in such already-selected subpopulation.

## MATERIALS AND METHODS

### Patients

A consecutive series of patients carrying a sensitizing mutation of the *EGFR* gene, receiving first line gefitinib were analyzed for mutations in 22 genes with deep sequencing technology. In all cases, a formalin-fixed and paraffin-embedded (FFPE) tumor excisional/trans-bronchial biopsy was available. Patients were retrospectively grouped according to time to progression during gefitinib treatment in: poor responders (progression at first tumor response assessment), intermediate responders (progression within 12 months), and good responders (progression or treatment still ongoing after 12 months). The principal aim of the study was to correlate the activity of gefitinib (in terms of treatment resistance and Progression-Free Survival, PFS) with: 1) the proportion of cells carrying the *EGFR* mutation, calculated on the basis of the value of the proportion of mutated alleles (PMA) that is derived from NGS analysis readout [Quantitative analysis], and 2) the presence of Additional Coexisting Mutations [ACM] (other than *EGFR* mutation) [Qualitative analysis]. All the samples were received anonymously and processed at the Molecular Pathology Unit of the Department of Pathology and Diagnostics of the University of Verona.

### Tissue microdissection and DNA preparation

Four 10-μm paraffin sections were manually microdissected to ensure that each tumor sample contained at least 60% of neoplastic cells. DNA was isolated using the QIAmp DNA FFPE tissue kit (Qiagen) and quantified and its quality assessed using NanoDrop® (Invitrogen Life Technologies; Milan, Italy) and Qubit® (Invitrogen Life Technologies) platforms [46, 49].

### Deep sequencing of multiplex PCR

Amplicons. Deep sequencing was performed using the Ion Torrent platform (Life Technologies). Briefly, 10 ng of purified genomic DNA were used for library construction with the Ion AmpliSeq Colon and Lung Cancer Panel v2 (Life Technologies) that targets 504 mutational hotspot regions of the following 22 cancer-associated genes, in alphabetical order: *AKT1, ALK, BRAF, CTNNB1, DDR2, EGFR, ERBB2, ERBB4, FBXW7, FGFR1, FGFR2, FGFR3, KRAS, MAP2K1, MET, NOTCH1, NRAS, PIK3CA, PTEN, SMAD4, STK11, TP53*. Emulsion PCR was performed either manually or with the OneTouch DL system (Life Technologies). The quality of the obtained library was evaluated by the Agilent® 2100 Bioanalyzer on-chip electrophoresis (Agilent Technologies; Santa Clara, CA). Sequencing was run on the Ion Torrent Personal Genome Machine™ (PGM, Life Technologies) loaded with a 316 chip as per manufacturer's protocol. Data analysis, including alignment to the hg19 human reference genome and variant calling, was done using the Torrent Suite Software v.3.2 (Life Technologies). Filtered variants were annotated using both the Ion Reporter software v1.2 (Life Technologies) and the SnpEff software v.3.0 (alignments visually verified with the Integrative Genomics Viewer; IGV v.2.1, Broad Institute) [46].

### DNA sanger sequencing

To validate NGS results, *KRAS* (exons 2, 3), *EGFR* (exons 19, 21), *TP53* (exons 5, 6, 7, 8), and *CTNNB1, PIK3CA, SMAD4* and *MET* specific PCR fragments were analyzed by Sanger sequencing. PCR products were purified using Agencourt AMPure XP magnetic beads (Beckman Coulter), labeled with Big Dye terminator v3.1 (Applied Biosystems). Agencourt CleanSEQ magnetic beads (Beckman Coulter) were used for post-labeling purification. Sequence analysis was performed on an Applied Biosystems 3130xl Genetic Analyser.

### Statistics

Descriptive statistics was used to summarize pertinent study information. Follow-up was analyzed and reported according to Shuster [50]. The correlation between variables were analyzed according to chi-square, Student's *t*, and Mann–Whitney (nonparametric) tests. The PMA value for each patient was normalized taking into account the rate of cellularity of malignant cells for each sample. The receiver operating characteristic (ROC) curve analysis was adopted for dichotomization of continuous variables according to outcome [51]. The hazard ratio (HR) and the 95% confidence intervals (95% CI) was estimated for each variable using the Cox univariate model [52]. Progression-free- and Overall-survival (PFS and OS) were calculated by the Kaplan-Meier product limit method from the date of treatment start until progression or death for any cause. The Log-rank and Tarone-Ware analyses were adopted to assess differences between curves. Significance was defined at the *p* < 0.05 level. The SPSS® (18.0) licensed statistical program was used for all analyses.

## SUPPLEMENTARY MATERIAL AND TABLE


