# Supplementary material for: Molecular heterogeneity assessment by next-generation sequencing and response to gefitinib of EGFR mutant advanced lung adenocarcinoma
Source: Oncotarget. 2015 Mar 30;6(14):12783–95. doi: 10.18632/oncotarget.3727 (PMC4494974; doi:10.18632/oncotarget.3727)
Supplement: Supplementary file 1 [file oncotarget-06-12783-s001.pdf]

Molecular heterogeneity assessment by next-generation sequencing and response to gefitinib of *EGFR* mutant advanced lung adenocarcinoma

Supplementary Material

Supplementary Table 1. Patients' characteristics according to NGS analysis: type of mutation and PMA.

Patient	Group	Type of Mutation (PMA)						
		EGFR	*TP53*	*KRAS*	*CTNNB1*	*PIK3CA*	*MET*	*SMAD4*
1	Good	L858R (32%)						
2	Good	p.E746_A750del (31%)						
3	Good	L858R (56%)						
4	Good	L858R (86%)						
5	Good	p.E746_A750del (71%)						
6	Good	p.E746_A750del (88%)						
7	Good	p.L747_T751del (64%)			S33C (20%)			G358E (44%)
8	Good	p.E746_S752delinsA (28%)			S45P (5%)			
9	Intermediate	p.L747QfsTer16 (23%)	R273G (45%)					
10	Intermediate	p.E746_A750del (94%)	R248W (48%)					
11	Intermediate	p.E745_A750del (30%)	R175H (27%)					
12	Poor	L858R (36%)	R273L (47%)					
13	Poor	p.E746_T751delinsIA (11%)		G12C (5%)				
14	Poor	p.E746_A750del (20%)						
15	Poor	L858R (43%)		G12C (22%)		E542K (31%)		
16	Poor	p.E746_A750del (31%)	R248L (40%)					
17	Poor	E746_A750del (94%)	R175L (37%)				N375S (34%)	

Legend – Supplementary Table 1. NGS: next generation sequencing; PMA: Proportion of Mutated Alleles,
